# Exploring the patterns of alpine vegetation of Eastern Bhutan: a case study from the Merak Himalaya

**DOI:** 10.1186/s40064-015-1066-8

**Published:** 2015-07-01

**Authors:** Karma Jamtsho, Kitichate Sridith

**Affiliations:** Department of Science, Nganglam Higher Secondary School, Nganglam, Pemagatshel 44102 Bhutan; Department of Biology, Faculty of Science, Prince of Songkla University, Hat Yai, Songkhla 90112 Thailand

**Keywords:** Vegetation, Eastern Bhutan, Himalaya, Alpine floristic community, Merak, Sakteng Wildlife Sanctuary

## Abstract

A survey was conducted from March to September 2012 along the altitudinal gradient of the Jomokungkhar trail in the Merak Himalaya of Sakteng Wildlife Sanctuary to study the floristic compositions and the patterns of alpine vegetation of Eastern Bhutan. The vegetation of the sampled plots is classified into five types of communities based on the hierarchical cluster analysis at similarity index 63% viz., (1) Riverine Community; (2) *Abies*–*Rhododendron* Woodland Community; (3) *Juniperus* Scrub Community; (4) *Rhododendron* Krummholz and (5) Alpine Meadow, based on the floristic compositions. In addition, it was noticed that the fragile alpine environment of the Merak Himalaya has high plant diversity and important plants that are susceptible to the anthropogenic pressures.

## Background

The Bhutan Himalaya is the main part of the Eastern Himalaya, which spans ca. 700 km (Oshawa 1987), located at the junction of two major biogeographic realms of the Indo-Malayan and the Palearctic. The region composed of mountains of simple slopes separated by deep river gorges and valleys consists of a number of unique habitats ranging from subtropical jungles in the south to alpine zones in the north. Although, the forests of Bhutan are still well managed under the strict rule of conservation policies, its success is likely to become a questionable issue owing to ever increasing pressures on natural environment from the increasing population and associated anthropogenic disturbances. Despite the remarkable efforts made and literatures available, very least is known about its flora and conservation strategies. The botanical studies of the recent past in the Bhutan Himalaya have focused mostly on the endemic, rare, and threatened plants or a particular taxon rather than the general biodiversity as mostly found in the Flora of Bhutan and some other works (Campbell and Long 1987; Clement 1999, 2001; Long and Rae 1991; Grierson and Long 1983, 1984, 1987, 1991, 1999, 2001; Grierson and Springate 2001; Gurung 2006; Gyeltshen 2012; Hoch 1991; Mill 1999, 2001; Pearce and Cribb 2002; Rae 1991; Wilson 1991). Therefore, floristic records of Bhutan may be incomplete and shall require further surveys and studies, especially in the remote and understudied areas. At present, Bhutan has 51.32% of its land covered with forests that are preserved through protected area systems and biological corridors (Nature Conservation Division 2001; World Wildlife Fund Bhutan and Sakteng Wildlife Sanctuary 2011).

Sakteng Wildlife Sanctuary (referred to as SWS hereon) was declared as a protected area of Bhutan in 2003. It is located in Trashigang district in the east of Bhutan and shares border with the adjacent Himalayan Range of Arunachal Pradesh in India. It is one of the protected areas in Bhutan with very rich biodiversity and amazingly houses ca. 35 species of *Rhododendron* L. (Wangchuk 2010) out of 46 recorded, so far, in the country (Pradhan 1999). Many other endemic, rare, and threatened plants also occur in SWS. In spite of the facts known, the previous account of botanical study in SWS is rather limited. Thus, the forests of these areas are under huge pressure from the local inhabitants and animal grazing. One ecologically important area of SWS is Merak: one of the remotest and most difficult areas to access for vegetation surveys and related research. The mountainous Merak has been also known to have the rich diversity of species of *Rhododendron* L. and many other endemic, rare, and threatened plants (Wangchuk 2010). In general, the vegetation of Merak is apparently composed of three types of forests: cool-temperate forest, subarctic forest, and dry alpine scrubs (Oshawa 1987; Grierson and Long 1983), with the major forest type composed of *Abies densa* Griff. This area is one among the scientifically unexplored areas, which is pressurized by anthropogenic disturbances. This work tried to evaluate the vegetation structure, composition and the distribution patterns along the altitudinal gradient ranging from the riverine forest of 3,320 m to the alpine meadow zone of 4,510 m of the Merak Himalaya, Trashigang district in East Bhutan.

## Methods

### The study area

**Figure 1:**
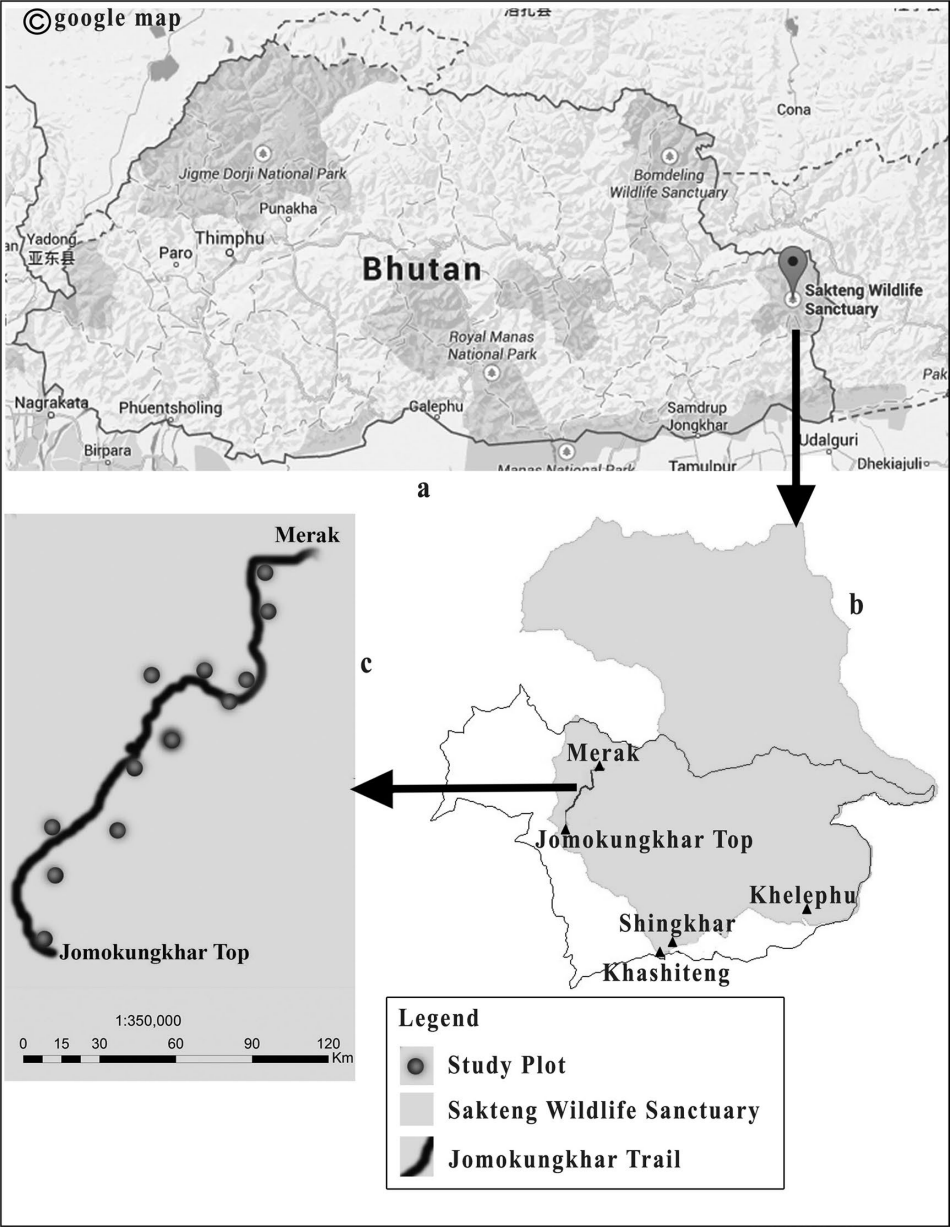
**a** Map of Bhutan showing SWS. **b** Map of SWS showing Merak and Jomokungkhar Trail. **c** Jomokungkhar trail and study plots.

Merak is located in Trashigang, the easternmost district of Bhutan. It lies within the preserved area of SWS (Figure 1a, b). The field survey was conducted along the altitudinal gradient of the *Jomokungkhar trail* (N27°17.762′–E091°50.268′ to N27°15.382′–E091°48.758′) of Merak. The trail is ca. 10.5 km in length, ranging from 3,320 m asl. (Ngera Ama river bank, Plot1; Table 1) to as high as 4,510 m asl. (Jomokungkhar top, Plot 12; Table 1). Within this range, various plant habitats, viz., woodland, scrubs, field layer, open ground, edges and aquatic terrestrial transitions are seen. The major type of forest is composed of coniferous woodland mixed with *Rhododendron* broadleaves and a few species of Junipers. However, it gradually changes to *Rhododendron* krummholz and to alpine meadow at the summit.

**Table 1:** Classification of study plots

Plot reference	Map reference	Elevation (m)	Site description
Plot 1	N27°17.762′ E091°50.268′	3320	Riverine forest
Plot 2	N27°17.532′ E091º50.142′	3420	Coniferous woodland
Plot 3	N27º17.017′ E091º50.069′	3520	
Plot 4	N27º16.956′ E091º49.882′	3620	
Plot 5	N27º17.029′ E091º49.801′	3720	
Plot 6	N27º17.096′ E091º49.693′	3820	
Plot 7	N27º16.965′ E091º49.367′	3920	Coniferous scrub
Plot 8	N27º16.878′ E091º49.311′	4020	
Plot 9	N27º16.473′ E091º49.047′	4120	Broad-leaves *Rhododendron* krummholz
Plot 10	N27º16.134′ E091º48.799′	4220	
Plot 11	N27º15.183′ E091º48.490′	4320	Alpine meadow
Plot 12	N27º15.387′ E091º48.758′	4510	

### Topography, geology and climate

The topography of Merak Himalaya is mountainous with East–West oriented upland valleys with varying degrees of slopes. The Merak Himalaya has an aggregate of mostly podzol and alpine meadow soils, found above 4,200 m asl (Okazaki 1987). With regard to the climate, Bhutan is the most unknown region in the monsoon Asia (Eguchi 1987). It has a wide variety of climatic conditions influenced by topography, elevation, and rainfall. It is divided into six climatic zones, viz. wet subtropical, humid subtropical, dry subtropical, warm temperate, cool temperate and alpine. Accordingly, the Merak Himalaya falls within alpine climatic zone with a mean temperature of 5.5°C and annual average rainfall of ≤650 mm (Department of Agriculture 1988). However, the paucity of information on monthly rainfall and temperature of the Merak Himalaya is due to the lack of meteorological stations within and in the adjacent areas.

### The study plots

Twelve study plots (Figure 1c; Table 1) were subjectively selected along the proposed transect line. Transect line was chosen in the least disturbed vegetation, which was assessed based on presence of alien species and anthropogenic disturbances. The homogenous continual natural condition and/or the presence and absence of natural remnant patches or characteristically unique floristic composition from the surrounding vegetation types were also included as plots selection criteria. In addition, various aspects of vegetation such as the presence of relict, pattern, and visual observation of species abundance were incorporated for choosing the plots. Table 1 depicts the characteristic properties of each study plot. The abundance of plant species was measured by cover-abundance estimation using the Domin-Krajina scale (Kent and Coker 1994). The numerical scores were recorded as follows (also see Table 2): + = a single individual, no measurable cover; 1 = 1–2 individuals with normal vigor, no measurable cover; 2 = several individual, but less than 1% cover; 3 = 1–4% cover; 4 = 4–10% cover; 5 = 11–25% cover; 6 = 26–33% cover; 7 = 34–50% cover; 8 = 51–75% cover; 9 = 76–90% cover; 10 = 91–100% cover.

**Table 2:** Cover-abundance estimation based on the Domin-Krajina scale

Family	Scientific name	Habit	Cover abundance											
			1	2	3	4	5	6	7	8	9	10	11	12
Monilophytes														
Davalliaceae	*Araiostegia* *faberiana* (C. Chr.) Ching	Fern (E)		2										
Dryopteridaceae	*Polystichum* sp.	Fern (T)		2										
Hymenophyllaceae	*Hymenophyllum polyanthos* Bosch	Fern (L)	2											
Polypodaceae	*Lepisorus contortus* (H. Christ) Ching.	Fern (E)	2											
	*Phymatopteris* *ebenipes* (Hook.) Pic. Serm.	Fern (E)	1											
	*Prosaptia* sp.	Fern (E)		2										
Eudicots														
Araliaceae	*Panax pseudoginseng* Wall.	Herb		1										
Asteraceae	*Anaphalis adnata* DC.	Herb	2											
	*Anaphalis nepalensis* var. *monocephala* (DC.) Hand.-Mazz.	Herb												1
	*Anaphalis* sp.	Herb	2											
	*Cicerbita* sp.	Herb	+											
	*Cremanthodium reniforme* (DC.) Benth.	Herb									+			
	*Ligularia fischeri* Turcz.	Herb	7											
	*Parasenecio* sp.	Herb			3									
	*Saussurea gossypiphora* D.Don.	Herb												2
	*Senecio raphanifolius* Wall. ex DC	Herb	8											
	*Soroseris hookeriana* Stebbins	Herb												2
Balsaminaceae	*Impatiens laxiflora* Edgew.	Herb	1											
Berberidaceae	*Berberis* *angulosa* Wall. ex Hook.f. & Thomson	Shrub								2				
	*Berberis* *virescens* Hook.f.	Shrub	2											
Boraginaceae	*Cynoglossum* *zeylanicum* (Lehm.) Brand	Herb	1											
	*Setulocarya* *diffusa* (Brand) R.R. Mill & D.G. Long	Herb	1											
Brassicaceae	*Cardamine griffithii* Hook.f. & Thomson	Herb	3											
Campanulaceae	*Cyananthus* *macrocalyx* subsp. *spathulifolius* (Nannf.) K.K. Shrestha	Herb										3	7	
Caryophyllaceae	*Arenaria* *densissima* Wall.	Herb											2	
	*Silene* *nigrescens* (Edgew.) Majumdar	Herb	3											
	*Stellaria* *sikkimensis* Hook.f.	Herb			1									
Celastraceae	*Parnassia* *chinensis* Franch.	Herb									2			
Diapensiaceae	*Diapensia himalaica* Hook.f. & Thomson	Herb											4	
Ericaceae	*Cassiope selaginoides* Hook.f. & Thomson	Herb							2	3				2
	*Gaultheria pyroloides* Hook.f. & Thomson ex Miq.	Herb							2	3				
	*Gaultheria trichophylla* Royle.	Herb							2	2				
	*Rhododendron* *anthopogon* var.*haemonium* (Balf. f. & R.E Cooper) Cowan & Davidian	Shrub									2			
	*Rhododendron* *argipeplum* Balf.f. & R.E.Cooper	Tree				3								
	*Rhododendron* *bhutanense* D.G. Long & Bowes Lyon	Tree								2				
	*Rhododendron campanulatum* subsp. *aeruginosum* (Hook. f.) D.F. Chamb.	Tree									5	10	3	
	*Rhododendron campylocarpum* Hook.f.	Tree	7											
	*Rhododendron cinnabarinum* Hook.f.	Tree					1							
	*Rhododendron fulgens* Hook.f.	Tree								6				
	*Rhododendron hodgsonii* Hook.f.	Tree				7								
	*Rhododendron kesangiae* D.G. Long & Rushforth	Tree			3									
	*Rhododendron lanatum* Hook.f.	Tree				4								
	*Rhododendron nivale* Hook.f.	Shrub						2						
	*Rhododendron setosum* D.Don	Shrub									2	2	7	8
	*Rhododendron thomsonii* Hook.f.	Tree						4						
	*Rhododendron* sp.	Tree							3					
Fabaceae	*Parochetus* *communis* D.Don	Herb	2	2	2	2	2	2						
	*Trifolium repens* L.	Herb	3	2		2	1							
Gentianaceae	*Gentiana capitata* Buch., Ham. ex D.Don	Herb	1											
	*Gentiana prostrate* var. *karelinii* (Griseb.) Kusn.	Herb		1			1	1						
	*Gentiana sikkimensis* C.B Clarke	Herb		2										
	*Halenia* *elliptica* D.Don	Herb	3											
	*Swertia assamensis* Harry Sm.	Herb									5			
Geraniaceae	*Geranium* *donianum* Sweet	Herb	2	2	1	1								
	*Geranium* *nepalense* Sweet	Herb	2											
Lamiaceae	*Clinopodium* *umbrosum* (M.Bieb.) Kuntze	Herb	2											
	*Phlomis* *tibetica* C.Marquand & Airy Shaw	Herb		+										
	*Prunella* *vulgaris* L.	Herb	2											
Onagraceae	*Epilobium* *gouldii* P.H.Raven	Herb	2											
Orobanchaceae	*Pedicularis* *siphonantha* D.Don	Herb	4	2	2	2								
Oxalidaceae	*Oxalis* *leucolepis* Diels	Herb	2	1	1									
Papavaraceae	*Corydalis* *crispa* Prain	Herb	2											
	*Meconopsis* *paniculata* Prain	Herb	+											
Plantaginaceae	*Hemiphragma heterophyllum* Wall.	Herb			1									
Polygonaceae	*Aconogonon campanulatum* (Hook.f.) Hara	Creeper				2								
	*Bistorta* *griffithii* (Hook.f.) Grierson	Herb								1	1			
	*Bistorta* *macrophylla* (D.Don) Soják	Herb	2	2		2	2							
	*Bistorta* *vacciniifolia* Greene	Herb										3		
	*Bistorta* sp.	Herb										1	3	
	*Persicaria* *nepalensis* (Meisn.) Miyabe	Herb	2											
	*Rheum acuminatum* Hook.f. & Thomson	Herb								2				
	*Rumex* *nepalensis* Spreng.	Herb	7											
Primulaceae	*Primula* *capitata* Hook.	Herb									2			
	*Primula* *deuteronana* Craib	Herb			2	2	2							
	*Primula* *dickieana* Watt, J. Linn. Soc.	Herb										2		
	*Primula* *gambeliana* Watt	Herb										2		
	*Primula glabra* Klatt	Herb			1						2	2		
	*Primula primulina* Spreng.	Herb									2			
	*Primula* sp.	Herb											2	
Ranunculaceae	*Anemone* *rupestris* Wall.	Herb						1						
	*Caltha* *palustris* L.	Herb				2								
	*Clematis* *montana* Buch.	Climber					1							
	*Delphinium viscosum* Hook.f. & Thomson	Herb	2											
	*Oxygraphis* *endlicheri* (Walp.) Bennet & Sum. Chandra	Herb	2	2	2	2	2	2	7					
	*Ranunculus* *brotherusii* Freyn	Herb	2											
	*Ranunculus* sp.	Herb	1											
Rosaceae	*Fragaria daltoniana* J.Gay	Herb	2		2									
	*Fragaria nubicola* (Hook. f.) Lindl. ex Lacaita	Herb	2											
	*Potentilla coriandrifolia* D.Don	Herb								2	2	4	1	
	*Potentilla* *cuneata* Wall. ex Lehm.	Herb	2											
	*Potentilla fruticosa* var. *arbuscula* (D. Don) Maxim.	Shrub									6			
	*Potentilla microphylla* D.Don	Herb											5	
	*Potentilla* *monanthes* var. *sibthorpiodes* Hook.f.	Herb	2											
	*Potentilla saundersiana* var. *caespitosa* (Lehm.) Th. Wolf	Herb								2				
	*Potentilla* sp.	Herb						2						
	*Rosa* *sericea* Lindl.	Shrub	1	1	1									
Saxifragaceae	*Bergenia* *purpurascens* (Hook.f. & Thomson) Engl.	Herb								2	2		2	
	*Saxifraga* *hispidula* D.Don	Herb									1			
Scrophulariaceae	*Oreosolen* *wattii* Hook. f.	Herb												2
Tamaricaceae	*Myricaria rosea* W.W.Sm.	Shrub	9											
Violaceae	*Viola* *biflora* L.	Herb	2											
Monocots														
Araceae	*Arisaema* *elephas* Buchet	Herb			1									
Juncaceae	*Jancus* sp.	Herb												1
	*Jancus* sp.	Herb												1
Liliaceae	*Lloydia* *flavonutans* Hara	Herb								2				
Orchidaceae	*Chusua pauciflora* (Lindl.) P.F.Hunt	Herb	2	2										
	*Satyrium nepalense* var. *ciliatum* (Lindl.) Hook.f.	Herb	2	2										
Pinopsida														
Cupressaceae	*Juniperus* *recurva* Buch.-Ham. ex D.Don	Tree	1	4					9	9		3	1	
	*Juniperus* *squamata* Buch.-Ham. ex D.Don	Tree		4					8	8		2		
Pinaceae	*Abies* *densa* Griff.	Tree		9	9	9	9	9	4	3				
	*Larix* *griffithii* Hook.f.	Tree	2											

*E* epiphytic, *L* lithophytic, *T* terrestrial.

Plant specimens from the study sites were collected once in a month, which commenced from March to September 2012, assuring full coverage of the flowering season for all plants. Some plant species occurring outside or in the adjacent areas of the study plots were also collected to obtain a complete checklist and precise pattern of diversity. The voucher specimens are deposited in the National Herbarium of Bhutan, Serbithang, Thimphu Bhutan, and the local herbarium of SWS, Department of Forest and Park Services (DoFPS), Ministry of Agriculture, Bhutan.

**Figure 2:**
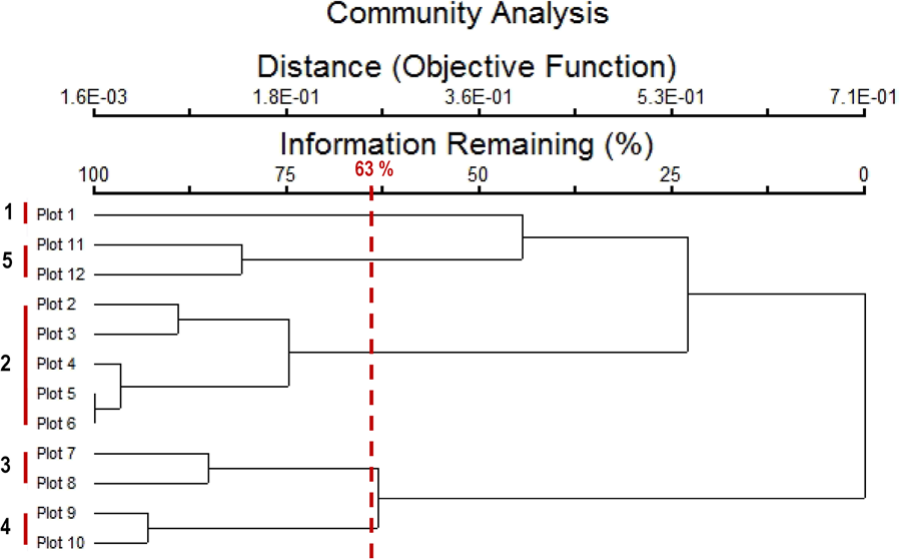
Cluster analysis dendrogram based on species abundances and habitats.

The Cluster Analysis was performed using PC-ORD software version 5.19 to classify the vegetation of the Merak Himalaya into different communities based on species abundances and habitats (Figure 2). The community types obtained from the analysis are described, photographed, and presented in profiles.

The floristic richness of the study areas was determined by the direct count of the number of species, genera, and families recorded. The graphical and tabular distribution pattern of a group of each life form and the overall distribution pattern of vascular plants of the study areas are presented.

## Results and discussions

### The vegetation patterns of the Merak Himalaya

The list of the plant species recorded and their abundance in each selected plot are shown is Table 2. Based on species abundance, twelve study plots identified along the line transect were grouped into community types by cluster analysis using PC-ORD. The hierarchical cluster analysis at similarity index 63% showed five types of communities (Figure 2; Table 3). The analysis was performed based on the species abundances and their habitats.

**Table 3:** Distribution pattern and species richness along the altitudinal gradient of the Merak Himalaya

Plots	Altitude (m)	Degree of slope	Vegetation	Number of species		
				Tree	Shrubs	Herbs
Plot 1	3,320	Flat	Riverine forest	3	6	17
Plot 2	3,420	Slope <45°	*Abies*–*Rhododendron* community	4	4	4
Plot 3	3,520			3	1	6
Plot 4	3,620	Slope >45°		2	1	4
Plot 5	3,720			1	2	3
Plot 6	3,820			1	1	5
Plot 7	3,920		*Juniperus* scrub Community	2		4
Plot 8	4,020	Slope ≤45°		1		7
Plot 9	4,120		*Rhododendron* krummholz		1	8
Plot 10	4,220				1	9
Plot 11	4,320	Slope >50°	Alpine meadow		1	9
Plot 12	4,510				1	10

**Figure 3:**
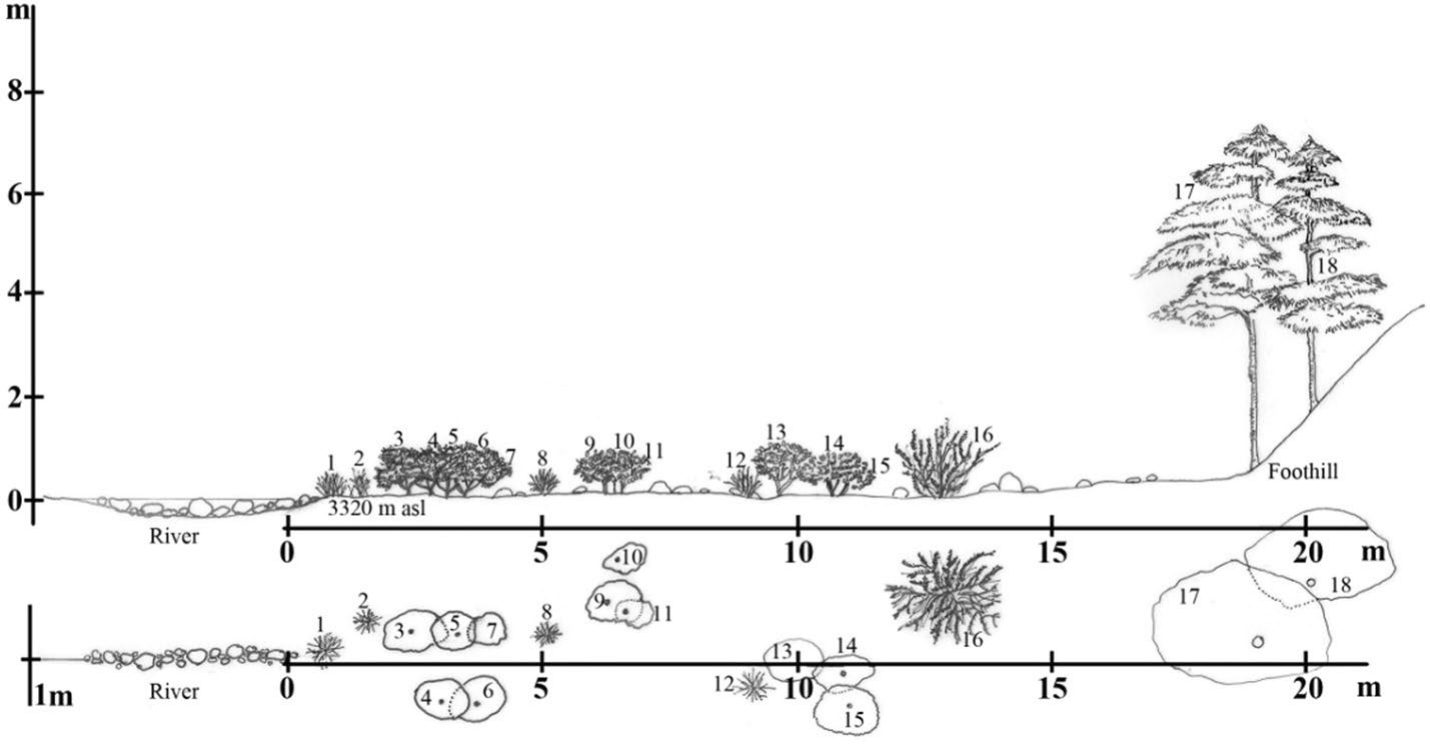
The vegetation profile the Riverine community of plot 1 (N27°17.762′; E091°50.268′) on the line transect of the Jomokungkhar trail: 1, 2, 8, 12—*Myricaria rosea* W.W.Sm; 3, 4, 5, 6, 7, 9, 10, 11, 13, 14, 15—*Rhododendron campylocarpum* Hook. f.; 16—*Berberis virescens* Hook. f.; 17, 18—*Abies densa* Griff.

**Figure 4:**
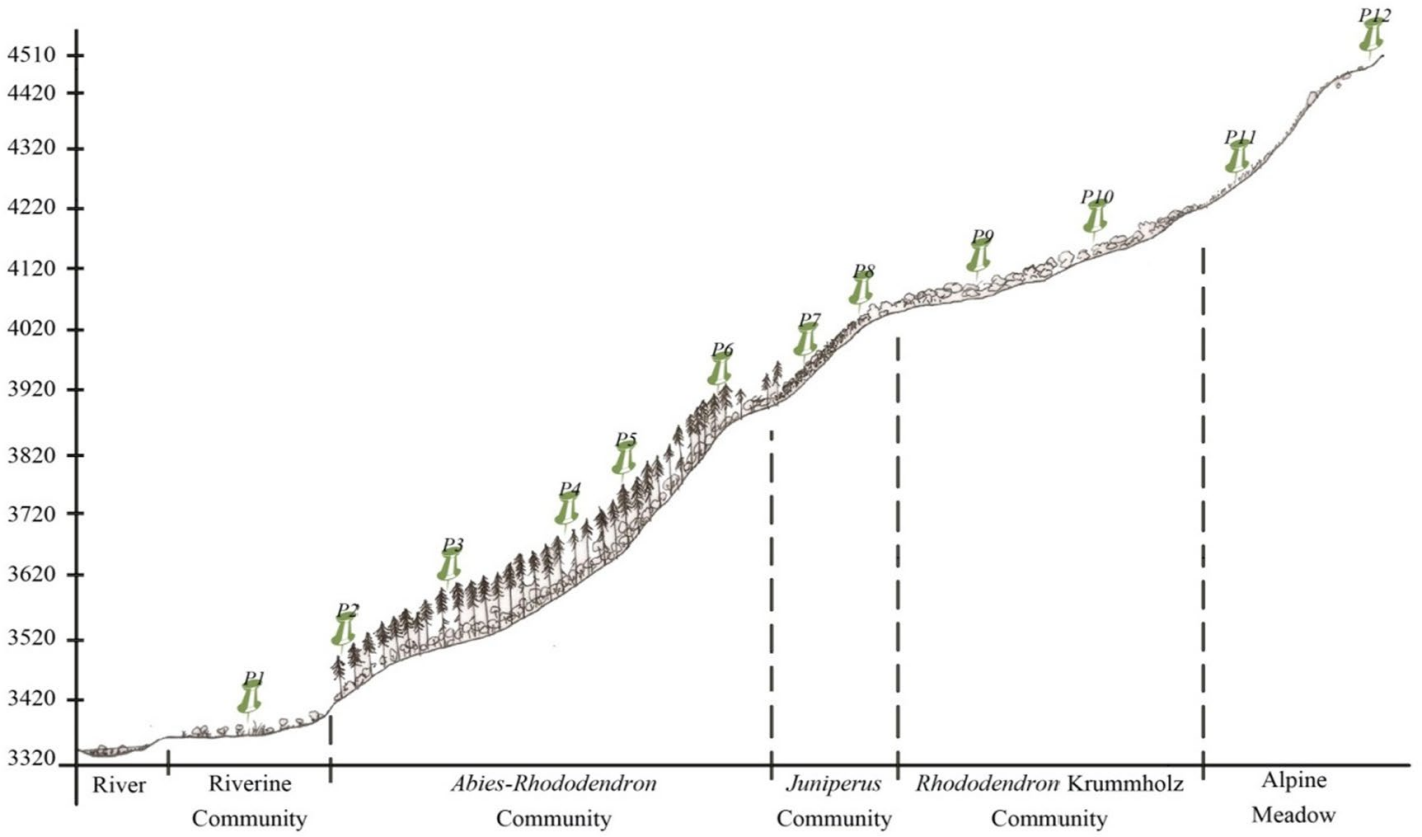
A schematic profile of vegetation pattern along the altitudinal gradient of the Merak Himalaya, Sakteng Wildlife sanctuary.

**Figure 5:**
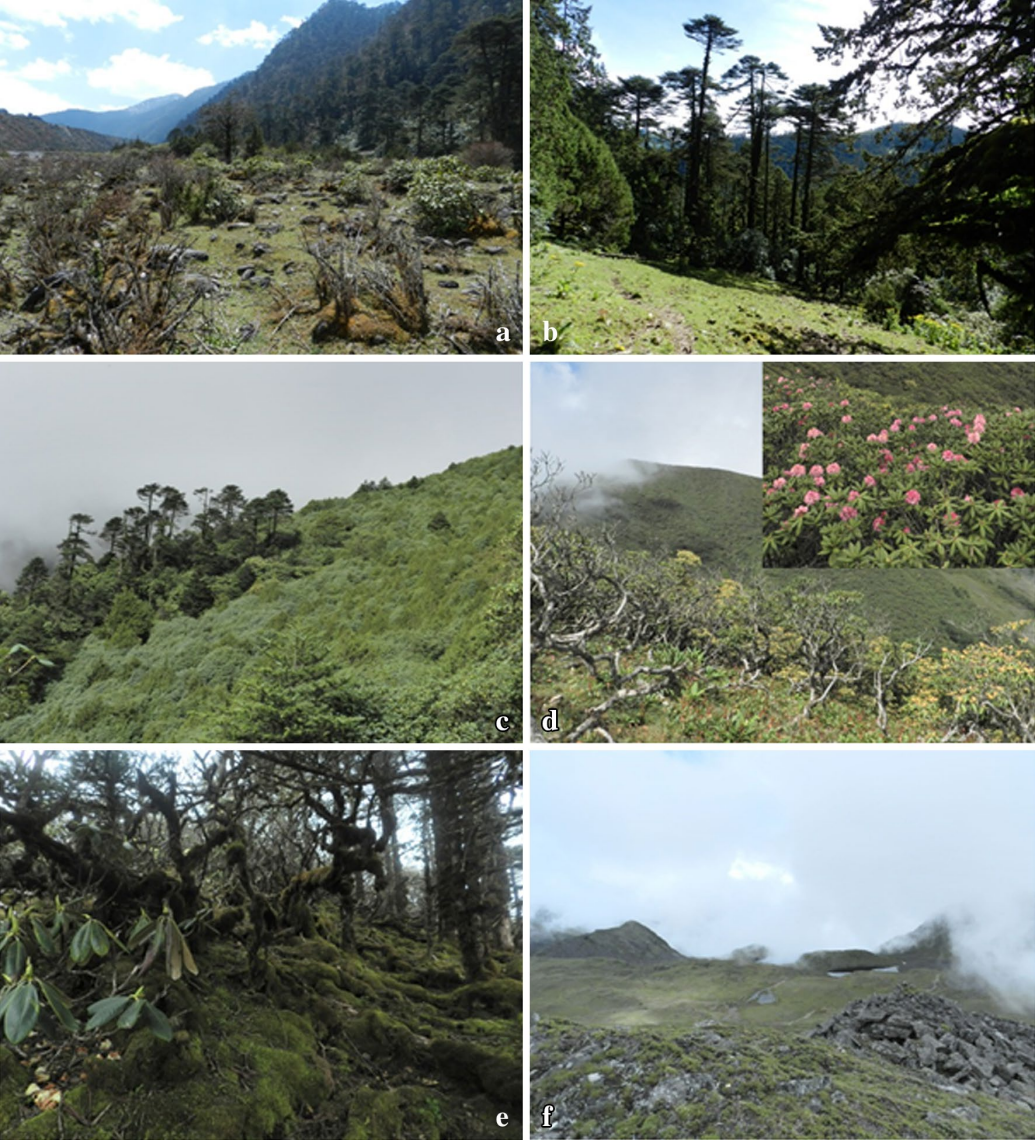
**a** Riverine community. **b** *Abies*–*Rhododendron* community. **c** *Juniperus* community. **d** *Rhododendron* krummholz. **e** Bryophyte covering ground floor of *Rhododendron* krummholz. **f** Alpine meadow.
  This type of community is rather restricted to the woodland habitat with sparsely occurring epiphytic ferns and dense bryophytes. It is mainly composed of a single tree species *A. densa* Griff—with high canopy and *Rhododendron* spp. as moderate occupants forming its understory vegetation (Figures 4, 5b, 6). Such vegetation is usually hilly with slopes of ±45° (Table 3). A very few species of shrubs like *Rosasericea* Lindl., *Juniperus* sp., *Rhododendron* sp., and *Berberis* spp.; and herbaceous plants like *Fragaria daltoniana* J. Gay, *Primula* spp., *Gaultheria trichophylla* Royle., and *Arisaema elephas* Buchet are seen on its ground floor.

1. Riverine community (Plot 1)
  Such type of community usually occurs alongside the river/streams (Figures 3, 4, 5a), where some of its portion experiences severe flooding during the heavy monsoon. It is composed of multiple microhabitats such as terrestrial scrubs, open ground, and aquatic-terrestrial transition. Most common terrestrial plants adorning such community are *Senecio raphanifolius* Wall. ex DC., *Rhododendron campylocarpum* Hook. f., *Rumex nepalensis* Spreng., *Pedicularis siphonantha* D. Don, and *Geranium* spp. *Myricaria rosea* W. W. Sm, *Cardamine griffithii* Hook. f. & Thomson and *Epilobium gouldii* P. H. Raven are some aquatic-terrestrial transition plants. *Fragaria nubicola* (Hook. f.) Lindl. ex Lacaita and *Anaphalis* spp. are among the herbaceous plants that occupy the open ground habitat.
2. *Abies*–*Rhododendron* Community (Plot 2–6)

**Figure 6:**
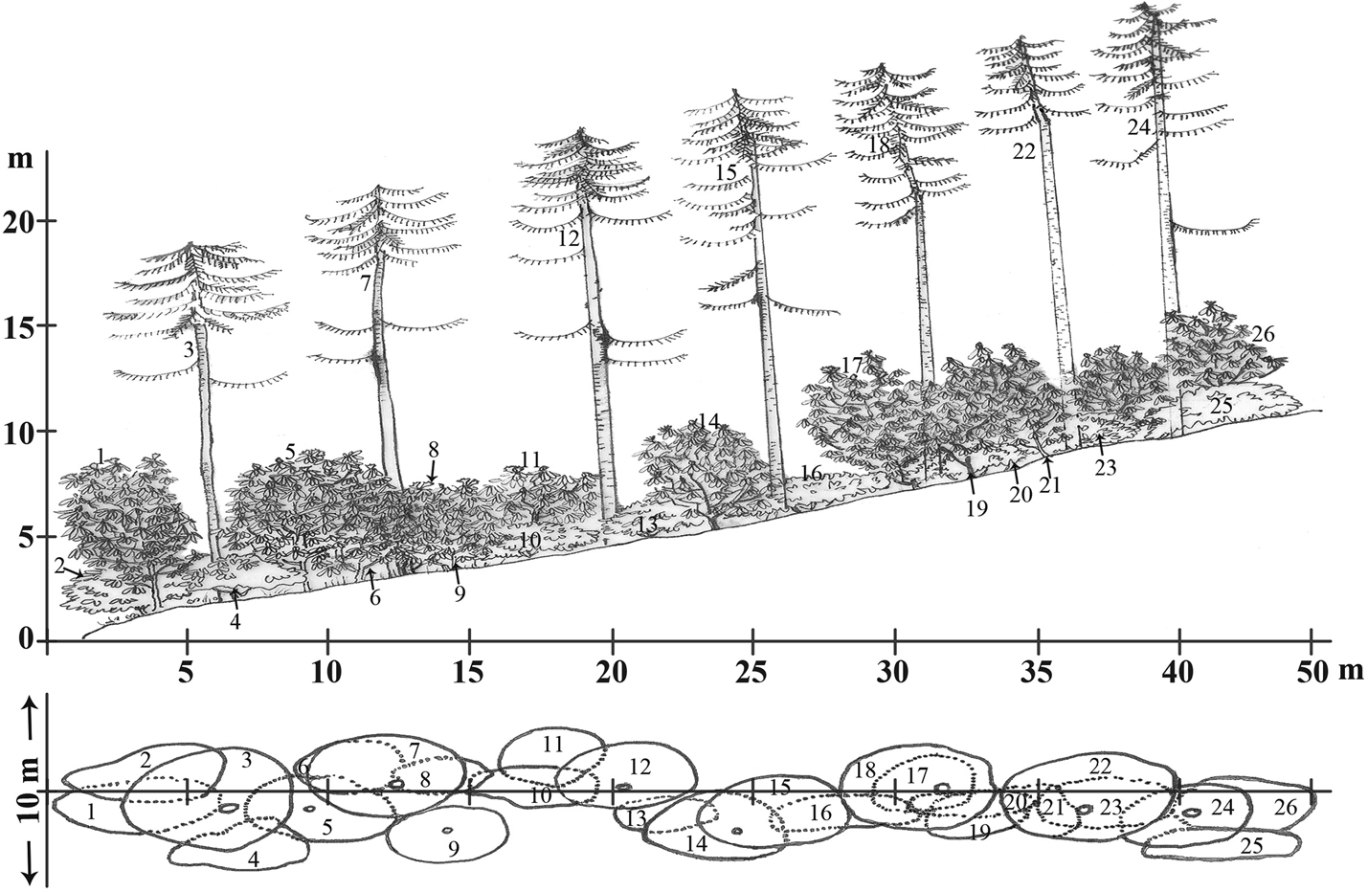
The vegetation profile of *Abies*–*Rhododendron* community of plot 3 on the line transect of the Jomokungkhar trail: 3, 7, 12, 15, 18, 22, 25—*Abies densa* Griff.; 2, 8, 19, 25—*Rhododendron argipeplum* Balf.f. & R.E. Cooper; 6, 11, 26, 23—*Rhododendron lanatum* Hook.f.; 9, 21, 26—*Rhododendron hodgsonii* Hook. f.; 4, 10, 13, 16—*Rhododendron fulgens* Hook. f.; 1, 5, 14, 17—*Rhododendron kesangaie* D.G. Long & Rushforth.
3. *Juniperus* Scrub Community (Plot 7–8)
  This is a small community forming above timberline vegetation (Figures 4, 5c). Mainly composed of *Juniperus recurva* Buch.-Ham. ex D. Don and *J. squamata* Buch.-Ham. ex D. Don, it is moderately to highly scattered vegetation (Table 3). *Gentiana* spp. and *Rhododendron nivale* Hook. f. are some other common species found in such community.
4. *Rhododendron* Krummholz Community (Plot 9–10)
  The *Rhododendron* krummholz is another type of community forming above timberline vegetation with a rich growth of bryophytes on its ground floor (Figure 5e). Krummholz here is referred to as the crooked and contorted forest formed by the effect of the wind current (Figure 5d). It is a fairly mixed vegetation of sparse and dense distribution of *Rhododendron* spp. Open grounds, gaps, and margins of such community usually have a high diversity of herbaceous vascular plants (Table 3). *Potentilla saundersiana* var. *caespitosa* (Lehm.) Th. Wolf, *Primula* spp., *Bergenia purpurascens* (Hook. f. & Thomson) Engl., *Oxygraphis* *endlicheri* (Walp.) Bennet & Sum. Chandra are some other taxa found in this type of community.
5. Alpine meadow Community (Plot 11–12)
  The Alpine Community (also see Figures 4, 5f) of the Merak Himalaya has a high diversity of herbaceous vascular plants (Table 3). This fragile natural mountain meadow is mostly adorned by the abundant growth of *Potentilla coriandrifolia* D. Don, *Bistorta* sp., *Cyananthus macrocalyx* subsp. *spathulifolius* (Nannf.) K. K. Shrestha, shrubby *Rhododendron setosum* D. Don, and numerous species of unidentified grasses.

The riverine community showed the richest diversity of herbaceous vascular plants (see Table 3) as compared to rest of the communities, despite the influence of anthropogenic disturbances. Some factors like soil quality, forest productivity, flat favorable topography, and overlapping of different plant assemblages forming an ecotone of evergreen coniferous forest and mixed broadleaved forest of the lower elevation region attribute for its rich floristic diversity.

The coniferous woodland, *Abies*–*Rhododendron* community, is the least species rich community (see Table 3). Adhikari (2005) reported that seedling establishment of the dominant species, *A. densa* Griff., of this community was thriving well under its own canopy with fairly distributed *Rhododendron* spp. in its understory. However, the phenomenon is not similar to other shrubs and herbaceous species. With the richness of epiphytic vascular plants dwindling with increasing altitude, edaphic factor and local topography of the community are other contributing factors.

The *Abies*–*Rhododendron* community of the Merak Himalaya has steep slopes of 45° or more (see Table 3). Such steep topography would then slow down the rate of seedling establishment and had made the soil poor through periodical leaching. Another apparent factor of rather low diversity in such community is the anthropogenic disturbance: animal grazing, tree felling, and deliberate burning of forest/individual species (also see Adhikari 2005). It is also expected that the high canopy (as high as up to 30 m) of *A. densa* Griff., and the broad-leaved *Rhododendron* spp. in its understory obstruct the light penetration to the ground, which is required by every plant for their successful growth and propagation.

On the other hand, the *Rhododendron* krummholz/scrubs of the Merak Himalaya provide special conditions for the diverse plant species to grow together in the same community through niche gapping. Therefore, this community also has a rich diversity of herbaceous plants along with rigorous growth of *Rhododendron* L. itself. *Bistorta griffithii* (Hook. f.) Grierson (Polygonaceae), *Potentilla saundersiana* var. *caespitosa* (Lehm.) Th. Wolf (Rosaceae), *Bergenia purpurascens* (Hook. f. & Thomson) Engl. (Saxifragaceae) flower in June. Within the same niche, other species of the same families drawing the same nutrients, e.g. *Rheum acuminatum* Hook. f. & Thomson, *Potentilla fruticosa* var. *arbuscula* (D. Don) Maxim., and *Saxifraga hispidula* D. Don flower in August. However, the pride of this community’s richness is counterbalanced by animal grazing. Palatable plants are grazed and unpalatable plants are trampled. In addition, it was noticed that there is higher species richness in the high alpine community than in the lower elevation communities. The alpine soil is characteristically poor in nutrients and significantly degraded easily by global climate change, grazing, human activities, and rodents (Wen et al. 2010). However, no mystical phenomenon was observed. The growth of herbaceous plants in the alpine community of the Merak Himalaya is well supported by less degree of anthropogenic disturbances and its topography. Slopes of this community are steep and over 45° where animals cannot access easily for grazing. There wasn’t any sign of impact to the environment by rodents. In the Himalayan Merak, human intervention in the succession of natural alpine environment is comparatively low as long as grazing practice is void.

A tremendous effort has been put by the management of Sakteng Wildlife Sanctuary to preserve the pristine natural environment of the region. Despite the strict regulations in place, grazing practices and trespassing of animal migrations can be seen around the “Jomophodrang” core zone. Such activities, if continued would pose threat to the fragile alpine ecosystem, where growth of many species is confined to a very small and restricted habitat. Total protection of the ecosystem would be difficult as long as the anthropogenic pressure persists.

## Endnotes

^a^Renewable Natural Resources.
